# IMPAACT 2016: Operationalizing HIV Intervention Adaptations to Inform the Science and Outcomes of Implementation

**DOI:** 10.3389/frph.2021.662912

**Published:** 2021-05-28

**Authors:** Jennifer L. Libous, Nicole A. Montañez, Dorothy E. Dow, Suad Kapetanovic, Janice Buckley, Tebogo Jacqueline Kakhu, Portia Kamthunzi, Limbika A. Maliwichi, Tichaona Vhembo, Tariro Dianah Chawana, Teacler Nematadzira, Geri R. Donenberg

**Affiliations:** ^1^International Maternal Pediatric Adolescent AIDS Clinical Trials Network, Science Facilitation, FHI 360, Washington, DC, United States; ^2^Division of Pediatrics Infectious Diseases, Duke University Medical Center, Durham, NC, United States; ^3^Department of Psychiatry and the Behavioral Sciences, University of Southern California, Los Angeles, CA, United States; ^4^Soweto International Maternal Pediatric Adolescent AIDS Clinical Trials Network Clinical Research Site, Johannesburg, South Africa; ^5^Gaborone Prevention/Treatment Trials Clinical Research Site, Botswana-Harvard School of Public Health-AIDS Initiative Partnership Clinical Trials Unit, Gaborone, Botswana; ^6^Malawi Clinical Research Site, University of North Carolina Project, Lilongwe, Malawi; ^7^College of Medicine-Johns Hopkins University Blantyre Clinical Research Site, Department of Psychology, University of Malawi-Chancellor College, Zomba, Malawi; ^8^Harare Family Care Clinical Research Site, University of Zimbabwe Clinical Trials Research Centre, Harare, Zimbabwe; ^9^St. Mary's Clinical Research Site, University of Zimbabwe Clinical Trials Research Centre, Chitungwiza, Zimbabwe; ^10^Seke North Clinical Research Site, University of Zimbabwe Clinical Trials Research Centre, Chitungwiza, Zimbabwe; ^11^Healthy Youths Program, Department of Medicine, Center for Dissemination and Implementation Science, University of Illinois at Chicago, Chicago, IL, United States

**Keywords:** adaptation, ADAPT-ITT framework, community engagement, intervention fidelity, implementation science, sexual health

## Abstract

**Introduction:** Uptake of evidence-based interventions for adolescents and young adults living with HIV (AYA-LWH) in sub-Saharan Africa (SSA) is complex, and cultural differences necessitate local adaptations to enhance effective implementation. Few models exist to guide intervention tailoring, yet operationalizing strategies is critical to inform science and implementation outcomes, namely acceptability, appropriateness, feasibility, fidelity, and sustainability. This paper describes operationalizing the ADAPT-ITT framework applied to a manualized trauma-informed cognitive behavioral therapy (TI-CBT) intervention addressing mental and sexual health for AYA-LWH in SSA in preparation for a randomized controlled trial (RCT).

**Methods:** Phase 1 of the RCT focused on operationalizing ADAPT-ITT steps 3–7 to tailor the intervention for use in eight sites across Botswana, Malawi, South Africa, and Zimbabwe. Well-defined processes were developed to supplement the general guidelines for each step to provide clear, consistent direction on how to prepare and conduct each step, including documenting, assessing, and determining adaptations, while maintaining intervention fidelity. The processes provided efficient standardized step-by-step progression designed for future replication. All sites participated in Phase 1 using the created tools and strategies to translate and present the TI-CBT to community stakeholders for feedback informing local adaptations.

**Results:** The research team developed and operationalized materials guiding adaptation. A translation review process verified local adaptability, maintained core concepts, and revealed differing interpretations of words, idioms, and culturally acceptable activities. Strategically designed tools comprised of feedback and translation verification forms resulted in meticulous management of adaptations. Robust collaborations between investigators, research managers, site personnel, and topical experts maximized multidisciplinary expertise, resulting in ~10–15 personnel per site facilitating, collecting, assessing, and integrating local feedback. Processes and tools operationalized in steps 3–7 effectively addressed implementation outcomes during community engagements (*n* = 108), focus groups (*n* = 5–8 AYA-LWH and caregivers per group), and strategic training of youth leaders.

**Discussion:** This paper offers a novel generalizable approach using well-defined processes to guide intervention adaptation building on the ADAPT-ITT framework. The processes strengthen the science of implementation and provide much-needed specificity in adaptation steps to optimize and sustain real-world impact and help researchers and community stakeholders maximize existing infrastructure, culture, and resources to inform implementation strategies.

## Introduction

The significant progress achieved to date in stemming new HIV infections has not benefitted everyone equally. Adolescents and young adults (AYA) (10–24 years of age) in sub-Saharan Africa (SSA) continue to experience disproportionate rates of new transmissions and suffer the highest mortality compared to all other groups (1), mainly as a result of high-risk sexual behavior (non-condom use, sex with multiple partners, early sexual initiation) (2). Rates of other sexually transmitted infections (STIs) are also elevated among AYA in SSA (3), underscoring the need for effective, efficient, and scalable sexual and reproductive health (SRH) programs for young Africans. Critically, AYA will soon constitute the majority population in many SSA countries, recently labeled the “adolescent bulge,” making SRH an urgent public health priority to ensure the long-term health of each nation (4).

Particularly germane to SSA is the absence of SRH services that are designed and available for AYA living with HIV (LWH) (5). As of 2020, over three-quarters of all AYA-LWH reside in SSA, and SRH efforts focused on this population have lagged behind other countries, in part because adolescent sexuality and sexual behavior in AYA is highly stigmatized, preventing open communication about safer sex options. SRH services are an essential lifeline to help AYA navigate healthy romantic and sexual relationships and family planning. The absence of effective SRH for AYA-LWH has been implicated in the forward transmission of infections.

The drivers of SRH for AYA-LWH are diverse and multi-level (6–8). One well-established determinant of SRH is mental distress, and 9–19% of adolescents (10–19 years of age) living with HIV reported elevated rates of mental health problems, including depression, anxiety, and trauma (9). Mental distress is implicated in poor ART adherence among AYA-LWH (10–12), minimal engagement in HIV care (13), and increased sexual risk taking, all of which contribute to low viral suppression and onward transmission of HIV (13). Despite the efficacy of ART in maintaining viral suppression, poor medication adherence among AYA-LWH has made viral suppression difficult to achieve. Addressing mental health problems is now considered an essential component of HIV care, but few evidence-based mental health services in SSA exist (9).

Health-care settings where AYA-LWH receive HIV services are ideal venues for both mental health and SRH programs (9), because they can be integrated into regular HIV care. Most SSA countries lack resources for AYA-focused SRH and mental health care (14, 15), namely adolescent-friendly services. Moreover, some SSA countries only have one or two psychiatrists for the entire country, and they are rarely focused on AYA seeking HIV related care (16). The number of trained mental health and SRH professionals is also highly limited, and thus, innovative approaches to service delivery that are sustainable and build local capacity to deliver mental health programming are urgently needed (17).

A seamless integration of HIV, SRH, and mental health care for AYA-LWH within health-care settings requires careful consideration and attention to implementation processes, barriers, and facilitators. Advanced planning for implementation will ensure that SRH and mental health programs are feasible, appropriate, acceptable, and sustainable at the patient, family, and provider levels. Such planning requires understanding the characteristics of the SRH and mental health programs (e.g., duration, cultural relevance), health-care setting factors (e.g., support for programs, personnel resources), staff attitudes and responsibilities (e.g., open to new learning, acceptance of programs), and individual youth and family expectations (e.g., SRH and mental health needs) that drive successful implementation. Similarly, preparing for implementation by vetting program content and logistics with key stakeholders and obtaining buy-in, providing rigorous training and supervision to program deliverers to ensure intervention fidelity, and close attention to reproducibility will facilitate successful implementation.

This paper outlines a systematic approach based on the ADAPT-ITT framework to adapting an evidence-based mental health intervention with attention to SRH for AYA-LWH and preparing for implementation in four countries in SSA. The *original source* of the intervention was *Trauma-Focused Cognitive Behavioral Therapy*, an empirically validated, resiliency-based mental health intervention that has successfully used psychoeducation to address the negative impact of stressful life or traumatic events in the United States, Asia, Africa, and Europe [see (18)]. The adaptation strategies outlined here build on the “Kigali Imbereheza Project” (KIP) meaning Bright Future, implemented in Kigali, Rwanda to develop and test a trauma-informed cognitive-behavioral intervention (TI-CBT) enhanced to address ART adherence and SRH for AYA-LWH. KIP engaged key Rwandan stakeholders (i.e., HIV care providers, mental health professionals, youth living with HIV, Ministry of Health officials) to assist in the adaptation of TI-CBT and strengthen the approach for 12–21-year-old Rwandans living with HIV. Using a systematic approach combining CDC guidelines (19) and the ADAPT-ITT model (20), the intervention was restated to be trauma-informed or TI-CBT noting the adaptations. KIP included six sessions: four of the original TI-CBT modules, and was enhanced with content examples and activities to directly apply cognitive-behavioral strategies to living with HIV and emphasize adherence to anti-retroviral medications for HIV. Outcomes of the 2-arm randomized controlled trial (R01HD074977) revealed statistically significant improvement in depression and anxiety in both arms, but the TI-CBT arm appeared to work slightly better for boys than girls. The absence of reliable biological data prevented examination of intervention effects on viral load or CD4 counts. Self-reported adherence, however, improved from baseline to 6 months in both conditions, but the improvement was not maintained at 18 months (21). For a full description of the process [see (22)].

The current paper takes the next step by further codifying the adaptation approach in preparation for a randomized controlled trial (RCT) by introducing systematic processes and documentation to facilitate tailoring and allow for replication and scale-up across four additional countries as part of the IMPAACT 2016 protocol. IMPAACT 2016 is part of the larger International Material Pediatric Adolescent AIDS Clinical Trials (IMPAACT) network whose mission is to significantly decrease incident HIV and HIV-associated infections and to decrease mortality and morbidity due to HIV and HIV-associated infections and co-morbidities among infants, children, adolescents, and pregnant/postpartum women. IMPAACT 2016, titled “Bright Future,” is designed to reduce mental health distress among AYA-LWH and in turn reduce ART nonadherence and poor virologic outcomes.

## Methods

### Design and Objective

This paper reports on Phase 1 of a multi-phase RCT that included the local adaptation and scale-up of a manualized TI-CBT intervention in SSA. The intervention consists of six 2 h group sessions delivered to AYA-LWH and two 2 h group sessions delivered to caregivers of AYA-LWH. A Youth Intervention Manual and a Caregiver Intervention Manual serve as scripts for delivering each session. The ADAPT-ITT framework guided the adaptation process and engaged local stakeholder feedback on acceptability, feasibility, appropriateness, and sustainability for successful implementation in low resource settings in SSA. The ADAPT-ITT framework (20) includes eight steps to systematically tailor evidence-based interventions as outlined in Table 1.

**Table 1:** Processes and tools developed to operationalize adaptation steps of the ADAPT-ITT framework to adapt the TI-CBT Intervention Manuals and Intervention Delivery.

**ADAPT-ITT step**	**Methods, processes, and tools**
**Step 1:** ***A**ssess a population's risk and protective factors*	• Identified important gaps in services for mental health problems, trauma, gender-based violence, sexual health, and barriers to ART adherence among 15–19-year-old youth living with HIV
**Step 2:** ***D**ecide on an intervention*	• Selected an evidence-based manualized TI-CBT intervention based on the KIP project, delivered by local peer youth and adult leaders within low resource settings of SSA
**Step 3:** ***A**dminister intervention components for local relevance feedback*	• Sites initiated translation and back-translation of the intervention manuals • Adaptation Team developed comprehensive adaptation feedback forms to collect, organize, manage, and document feedback • *Community Stakeholder Engagements*: Sites solicited feedback on session activities, including, strengths and weaknesses, clarity, feasibility, acceptability and barriers, and component(s) needing improvement that might benefit from focus group testing • *Focus Groups*: Per feedback from community stakeholder engagements, sites delivered selected session activities to youth and caregivers for additional feedback
**Step 4**: ***P**roduce intervention modifications*	• Adaptation Team reviewed adaptation feedback forms on a rolling basis • Feedback was categorized for single-site adaptation vs. cross-site adaptation
**Step 5:** ***T**opical expert consultations*	• Topical experts were available for consultation as needed, including site personnel to clarify cultural appropriateness
**Step 6:** ***I**ntegrate feedback into intervention*	• Adaptation Team integrated modifications applied to all sites into manuals • Sites integrated site-specific modifications for their individual manuals as needed • Sites translated and back-translated new content as needed
**Step 7:** ***T**rain personnel delivering intervention*	• Standardized criteria were used to select group facilitators and supervisors to oversee intervention delivery • Expert Trainers trained youth leaders, adult study staff, and supervisors over 2 weeks in-country • Instituted a cascading supervision model to ensure sustained intervention fidelity
**Step 8:** ***T**est modified intervention*	• Modified intervention manuals will be pilot-tested to fine-tune and practice the logistics of the intervention at each site

Steps 1 and 2 of the ADAPT-ITT framework are generally exploratory to assess a population's risk factors and inform a decision to select and adapt an appropriate intervention. This paper details steps 3 to 7 of the ADAPT-ITT framework, which are designed to adapt an intervention, and provides clear recommendations for operationalizing and documenting adaptations. Strategically operationalizing the ADAPT-ITT framework had the following objectives: 1) develop processes and tools to standardize the adaptation steps of the ADAPT-ITT framework and allow for replication and scale-up; 2) confirm local acceptability, feasibility, appropriateness, and sustainability; and 3) systematically document local feedback to synchronize adaptation procedures across populations while maintaining intervention fidelity.

### Setting and Population

Eight clinical research sites (CRSs) in the IMPAACT Network across four countries in SSA were selected to participate in this study: Soweto IMPAACT CRS in Soweto, South Africa; Gaborone Prevention/Treatment Trials CRS in Gaborone, Botswana; Molepolole Prevention/Treatment Trials CRS in Molepolole, Bostwana; Malawi CRS in Lilongwe, Malawi; College of Medicine Johns Hopkins University CRS in Blantyre, Malawi; Harare Family Care CRS in Harare, Zimbabwe; Seke North CRS in Harare, Zimbabwe; and St Mary's CRS in Chitungwiza, Zimbabwe. Selection criteria included 1) lack of adolescent and young adult-focused SRH and mental health care infrastructure (e.g., minimal or no mental health screening and counseling, trained professionals, and support services), 2) sufficient volume of AYA-LWH to enroll in the study, and 3) availability of young adults ages 21–30 years old living with HIV who could be trained to deliver the TI-CBT intervention.

### Procedures

The adaptation process was led by a multidisciplinary Adaptation Team including investigators, medical professionals, and clinical researchers with collective experience in behavioral and implementation science, psychology, psychiatry, intervention adaptation, research management, and/or clinical care of AYA-LWH in SSA and in collaboration with site personnel. Local community stakeholders were invited to participate in meetings and/or subsequent content specific focus groups (one for AYA-LWH and one for caregivers of AYA-LWH) to provide feedback about the intervention content and relevance. Prior to stakeholder engagement meetings, the intervention manuals were translated and back-translated into local language(s) as needed. The Adaptation Team reviewed the community stakeholder feedback and the back-translations to confirm core elements were retained.

#### Step 3: Local Community Engagement for Intervention Feedback

Intervention feedback was solicited through a two-fold approach: community stakeholder meetings followed by targeted focus groups. Stakeholder meetings included community and youth advisory boards, counselors, nurses, and others who met with study site personnel to review the intervention content and provide feedback. Study site facilitators discussed each intervention activity and then requested feedback on its specific strengths and weaknesses using open-ended questions, as outlined in Supplementary Figure 1. Adaptations were recommended and components that could be challenging to deliver in the local context were identified for further evaluation in subsequent focus groups.

The Adaptation Team held an in-person training with study site personnel to review core intervention concepts and activities. Additionally, the Adaptation Team provided video-recordings of activities for sites to reference in preparation for the site focus group. Focus group participants were 15–19-year-old AYA-LWH, and caregivers were adults who cared for AYA-LWH. Facilitators presented targeted intervention activities identified by stakeholders as challenging and requested feedback, while site staff observed and documented participant reactions. Discussions emphasized each component's acceptability and relevance, as outlined in Supplementary Figure 2.

#### Steps 4 and 5: Reviewing Feedback for Production and Topical Experts in the Adaptation Team

Feedback forms used in the stakeholder meetings and focus groups were designed to efficiently guide, document, manage, and categorize local feedback. As illustrated in Supplementary Figures 1, 2, page 1 summarized the feedback, and the following pages prompted recommendations regarding clarity, feasibility, acceptability, relevance, and barriers to understanding content and intervention delivery. Each category was accompanied by a table for facilitators to organize and document notes summarizing feedback source (e.g., non-identifiable participant demographics such as age, role, and sex) and the associated feedback.

Feedback forms were reviewed by the Adaptation Team on a rolling basis, with all stakeholder engagement activities completed at the site prior to the focus groups. Inductive coding identified topics and themes in the feedback, and these were categorized as site-specific or relevant across sites. Final adaptations ensured that core intervention concepts and constructs were not altered in order to maintain intervention fidelity, and these were documented on each site's adaption feedback form.

#### Step 6: Integrating Feedback as Adaptations

The Adaptation Team integrated the modifications into the Youth Intervention Manual and Caregiver Intervention Manual to ensure consistency. As needed, sites translated and back-translated new content into the local language.

#### Step 7: Training

Standardized criteria were used by sites to choose peer youth leaders and adult study staff to deliver the intervention. Peer leaders were age 21–30 and living with HIV, and adult study staff had experience in mental health or HIV counseling. Local supervisors with a background in mental health or clinical experience supervised youth and adult study staff to ensure intervention fidelity. The training was guided by a training manual adopted from KIP (23), and involved a lead Expert Trainer, four members of the Adaptation Team (who were trained to become Expert Trainers for scale-up in other countries), two site supervisors, four peer youth leaders, and three adult study staff. The training occurred over 2 weeks, ~6–8 h each day. The first 3 days focused on the underlying theory and concepts associated with TI-CBT. The remaining 7 days emphasized content delivery, practice, and feedback. Adult study staff received 4 h of training over 2 days focused on content delivery, practice, and feedback. All trained facilitators continued to practice with local supervisors until deemed competent.

## Results

### Outcomes of Operationalizing Adaptation Steps

The Adaptation Team met weekly to develop standardized materials to guide the adaptation processes in Steps 3–7 outlined in Table 1. All sites used the forms to systematically document participant feedback regarding the strengths and weaknesses of the intervention and local feasibility, acceptability, clarity, and relevance. Robust collaborations between investigators, research managers, site personnel, and topical expert consultants maximized multidisciplinary expertise, and resulted in ~10–15 personnel per site collaborating to effectively facilitate, collect, assess, and integrate local feedback in preparation for the RCT.

Progression through the adaptation steps varied across sites due to differing local regulatory requirements, translation needs, and site-specific resources (e.g., personnel). While the ADAPT-ITT framework implies a somewhat linear approach to adaptation, this paper presents a more cyclical workflow during Steps 4–6. The continuous feedback loop allowed for ongoing adaptation feedback, review, production, and integration processes as sites progressed at different paces and additional feedback received during the training required integration.

The translation and back-translation verification process was time-intensive but a crucial informative part of the adaption steps that contributed to varying paces. Interestingly, each site chose to conduct the community engagements in English, yet they also translated and back-translated the manuals to ensure local understanding and address diverse literacy needs. The Adaptation Team carefully tracked and monitored the manual translations (and back-translations) for accuracy. The study employed three key structured manuals: one for the youth sessions, one for the caregiver sessions, and one that guided training. The two intervention manuals were translated into five languages: Zulu and Sesotho in South Africa, Setswana in Botswana, Chichewa in Malawi, and Shona in Zimbabwe.

The translation and back-translation verification process confirmed core concepts and constructs were maintained, yet revealed differing interpretations of words, idioms, and culturally acceptable activities. Importantly, language-specific anatomical, cognitive, and behavioral concepts and terms were back-translated differently compared to English, and these were documented on a verification form. These differences prompted consultation with site personnel for clarity and confirmation that the translation was appropriate for the intended concept or term. The consistent back-and-forth communication between the sites and the Adaptation Team required patience, flexibility, open-mindedness, and collaboration.

### Outcomes of Local Community Engagement and Intervention Feedback

A total of 108 individuals across the eight sites participated in the community stakeholder engagement activities, representing males and females, a wide age range, diverse roles within the communities, and different race/ethnicities within countries. As summarized in Table 2, an average of 13 community members per site spanning 16–83 years old, with over 50% females, participated across all sites. Diverse community roles were represented with Community Advisory Board members from each site and additional roles spanning hospital and clinic staff, mental health, social workers, counselors, youth, and Ministry of Health. Broad engagement across demographic groups provided valuable insight regarding perceptions by individual stakeholders (youth and adults) across sites.

**Table 2:** Demographics of participants in community stakeholder engagements.

	**South Africa**	**Botswana**	**Zimbabwe**	**Malawi**	
				**Blantyre**	**Lilongwe**
Sample size (*n*)	11	32	33	19	13
Female (%)	73	50	83	63	54
Age range (years)	16–83	16–60	18–69	18–62	17–30 and over
Represented Roles^*^	Adolescent CAB Treatment CAB	CAB Site staff Caregivers Hospital staff Counselor Social Welfare Adolescent student Ministry of Health Support group staff	Adult CAB Youth CAB Study staff HIV counselors AYA-LWH	CAB Teen club youth Psychiatric nurse Research nurse Study coordinator Clinic manager Educator	Youth CAB Mental health nurse Mental health worker Study staff

* *CAB: Community Advisory Board*.

Sites within Botswana and Zimbabwe felt they shared language and culture within their respective countries such that only one AYA-LWH focus group (*n* = 5–8) and one caregiver focus group (*n* = 5–8) per country was needed. The sites within Botswana and the sites within Zimbabwe determined who would host the focus groups and collaborated in the preparations and conduct. By contrast, the two sites in Malawi were deemed considerably different, necessitating separate focus groups for each site [one for AYA-LWH (*n* = 5–8) and one for caregivers (*n* = 5–8)]. Participants in the focus groups represented the AYA-LWH and caregiver priority populations. Focus groups sought feedback on the intervention's acceptability and relevance. Prior to COVID-19, South Africa and Botswana completed their focus groups, but operational disruptions from the COVID-19 pandemic have required postponement of focus groups in Malawi and Zimbabwe to safeguard participants and align with local guidelines.

Focus group and stakeholder feedback was placed into three categories: 1) required modification to the intervention manuals for *all sites* (per Step 6), 2) optional modification at a *specific site* (per Step 6), or 3) clarification via written feedback to the site or during facilitator training (Step 7). As summarized in Table 3, examples of feedback resulting in required manual adaptations across all sites to maintain consistency and core constructs were integrated by the Adaptation Team. Examples of feedback resulting in optional manual adaptations were to be made by the local sites to allow for cultural relevance, acceptability, and feasibility.

**Table 3:** Examples of key feedback resulting in required vs. optional manual adaptations across sites.

**Topic and feedback**	**Feedback received from^1^**	**Required vs. optional manual adaptations across sites**
**SEXUAL HEALTH**		
• Add female condom and demonstration	E, F, G, H	Required for all sites
• Add guidance on checking the condoms expiry date on the packet and how to determine if a condom looks and feels useable	F, G, H	Required for all sites
• Add option for mixed vs. separate sex/gender groups to discuss relationships and/or condom demonstration or sex education	A, E	Optional for A and E
• Clarify that group leaders may encourage AYA to choose their identified-gender group	B, C	Optional for B and C
• Ensure proper translations of sensitive topics such as sexual behavior to retain meaning	A	No change
• Note that a woman is expected to submit to the husband's sexual needs even if she does not want to	D	No change
**LOGISTICS**		
**Emphasis on Confidentiality:**		
• Reinforce to participants the importance of confidentiality risks	A	Required for all sites
• “Make it clear to the teens that they should not share the information of sessions to teen clubs including social media.”	D	Required for all sites
• Clearly explain confidentiality. Differentiate it from ‘keeping things to self' vs ‘keeping study and session proceedings within the group' and ‘confidentiality' vs ‘stigma (participants failing to disclose issues due to fear of stigma)'. Create a “confidentiality statement/log” for all staff, facilitators, and participants to sign.	B, C	Required for all sites
**Allow more time during a session for specific topics:**		
• Add time for cognitive triangle (connection between thoughts, feelings, and behavior) and relationships	A, F, G, H	No change
• Add time for the body awareness activity increasing from 20 to 40 min	D	No change
**CULTURE**		
• Clarify that sites should assign partners for the body mapping activity to ensure same-gender identified pairs	D, F, G, H	Required for all sites
• Clearly explain the meaning of depression: “Culturally, depression and ‘stress' may be perceived as similar.”	A	No change

1 *Applicable sites identified as follows: A (Soweto IMPAACT CRS), B (Gaborone Prevention/Treatment Trial CRS), C (Molepolole Prevention/Treatment CRS), D (Malawi CRS), E (College of Medicine Johns Hopkins University CRS), F (Harare Family Care CRS), G (Seke North CRS), and H (St Mary's CRS)*.

Examples of required manual adaptation across all sites pertinent to SRH included feedback about activities involving physical contact between males and females and new female condom information. Stakeholders recommended that youth be paired with the same sex/gender for close contact activities such as the body mapping component, where youth trace the outline of another youth's body, and the inclusion of a female condom demonstration and discussion to avoid gender bias and support female-controlled HIV prevention methods.

Examples of optional site-specific manual adaptations included feedback regarding activities involving sensitive topics about relationships, condom demonstrations, and sex education. Stakeholders recommended AYA-LWH choose whether to conduct these activities in mixed vs. same sex/gender groups and that AYA-LWH who identify as neither female nor male may select which group to join to feel included, comfortable, and safe.

Focus group participants recommended other additions to the curriculum. The South African site suggested adding content to help AYA-LWH disclose their HIV status to partners. They also advised splitting the group by sex/gender to discuss sexual health and demonstrate condom use. Feedback from the South African caregiver focus group suggested new content to empower and help caregivers cope with feelings of guilt. Interestingly, Botswana's site feedback focused on the importance of caregiver HIV disclosure to youth, the ages of youth when discussing sexual health, and suggested emphasizing ART adherence through added HIV treatment education.

Some feedback did not require manual adaptations, but rather was effectively addressed through clarification or during facilitator training. Stakeholders were concerned that sensitive topics and key concepts might get lost in translation (i.e., depression, stress, and sexual behavior), and these were addressed during the concurrent translation and back-translation verification process to ensure meanings were accurately conveyed in the local language and context. Stakeholders also identified topics they felt warranted more discussion time, such as violence, relationships, thoughts, feelings, and behaviors. Sites were able to adjust the amount of time spent on activities to ensure youth understanding, and all feedback was provided during training to help facilitators navigate different topics and discussion scenarios.

The adaptation forms were vital low-tech tools used across sites and the Adaptation Team to operationalize the ADAPT-ITT steps and improve the cultural feasibility, acceptability, and relevance of the intervention manuals. The forms provided a seamless process that linked each adaptation step to its outcome. They efficiently documented feedback during community stakeholder engagements and focus groups (Step 3); facilitated timely review of feedback and a clear plan for adaptation (Steps 4–5); and directed the adaptations (Step 6). The forms were further available to guide on-site trainers and identify intervention components that proved challenging in the local context and required clarification during training (Step 7). Throughout Steps 3–7, careful attention was paid to ensure that content believed to drive behavior change (core elements) was not altered. The forms provided a systematic approach to integrate adaptations into the revised manuals. All adaptations were included in a summary of modifications table that described the type (e.g., language or activity) and session number. This methodology preserved version control across all sites.

### Outcomes of Training

For the first hour, the expert trainer presented the material/activity/component to the trainees, during which the participants noted any areas that needed further clarity. These were documented as needed revisions to the manuals. Next, the trainees conducted the activities for practice and feedback, and this was an additional opportunity for modifications and clarifications; most of these increased facilitation guidance, provided section rewording, and added detail in the directions for clarity on activities. All changes to the manual were implemented in real-time while trainees practiced delivering the intervention. In some cases, additional content was integrated to address gaps in the intervention (e.g., female condom use). The training strategically maximized existing local infrastructure and resources (e.g., capable local youth and adults) to further build capacity in country to deliver a SRH and mental health intervention in the low resource setting.

## Discussion

There remain substantial gaps in adapting evidence-based interventions into practice, particularly in low resource settings across SSA where access to SRH and mental health services for AYA-LWH are limited. These gaps reflect numerous factors including a lack of trained medical professionals and interventions not designed appropriately for the existing infrastructure, culture, or age. The Adaptation Team members from the IMPAACT 2016 project sought to address such gaps in rapid succession across multiple locations by establishing well-defined, comprehensive processes, and tools to operationalize the ADAPT-ITT framework for community-based intervention adaptation.

The ADAPT-ITT framework is instrumental in systematically adapting interventions, and application of the framework can vary depending on the intervention, population, and resources (24–26). Significant advancements have been made in developing guidelines on how to apply the ADAPT-ITT framework on a small scale (24, 25), and there is growing interest for further advancing the application of the ADAPT-ITT framework to a larger scale. Supplementing the general guidelines of the ADAPT-ITT framework with specific operationalization plans outlining systematic processes for each adaptation step offers a robust strategy (20, 25) to tailor the framework and replicate for future scale-up of various types of interventions.

By operationalizing the ADAPT-ITT framework, we were able to the use the voice of the community to produce and integrate important adaptations and create a best fit intervention addressing mental health and SRH needs of AYA-LWH across four countries in SSA. It was important to not limit stakeholder demographic to one specific group (e.g., decision makers or priority population) so as to benefit from multiple perspectives and fully contextualize feedback. Moreover, continuous efforts to gather feedback allowed for rapid identification of concerns with stakeholders followed by focused engagement with the priority population during focus groups to confirm adaptations best suited for the existing infrastructure. Feedback informed modifications to the intervention manual with additional guidance on female condom use, HIV disclosure, gender composition during close contact activities or discussing relationships, and age of youth discussing sexual health to make more culturally relevant and acceptable. Yet, the degree of content change was less than anticipated which was valuable to confirm through the ADAPT-ITT process to ensure effort and costs invested in future large-scale implementation are warranted.

Operationalizing the ADAPT-ITT framework to allow for intervention adaptation across multiple sites concurrently supports rapid scale-up but has layers of intertwining complexity to carefully coordinate depending on if integrating cross-site or site-specific and the need to translate intervention manuals. When preparing an adaptation operationalization plan and timeline, we recommend the use of check points and pauses to allow for different-paced sites to benefit from each other's feedback and adaptations. We also recommend initiating the translation and back-translation verification process as early as possible during Step 3. To the extent possible, incorporating the translations within the community engagements will maximize local feedback to efficiently address concepts, words, and idioms that are challenging to deliver in a different language and prevent losses in translation.

The application of the ADAPT-ITT framework on a large scale has its limitations. It requires significant personnel time across participating sites and teams to collectively ensure appropriate and relevant tailoring to the diverse needs of existing infrastructures as well as to maintain fidelity to ensure the best mental and SRH outcomes. While there is a significant upfront time cost, it allowed for cross-site adaptations, and as a result, we anticipate a more sustainable intervention applicable to multiple SSA settings. The investment is likely to have long-term impact by improving SRH, increasing viral suppression, and reducing mental health distress among AYA-LWH. Additional limitations came from operational disruptions due to the COVID-19 pandemic. Our community engagement approach was not designed for virtual implementation, and instead relied on in-person interactive engagements. Consequently, intervention delivery across sites is postponed and will require re-training.

Upon resuming operations, sites will proceed with training, focus groups, and then pilot testing the adapted TI-CBT intervention in preparation for the RCT. It is our hope that the processes and tools developed and presented here will help guide future adaptations. The next step will be to ensure sustainability of these interventions through capacity building (as we propose here using youth peer leaders to deliver the program) and through rigorous supervision models to maintain fidelity and reproducibility (22).

## Conclusion

The systematic approach described in this paper strengthens the science of implementation for scale-up efforts and provides much-needed specificity in adaptation steps to optimize sustained real-world impact on mental health and SRH in SSA. This specificity allows researchers and community stakeholders to maximize existing infrastructure, culture, and resources to inform implementation strategies to achieve local acceptability, appropriateness, feasibility, fidelity, and sustainability.

## Data Availability Statement

The raw data supporting the conclusions of this article will be made available by the authors, without undue reservation.

## Ethics Statement

The studies involving human participants were reviewed and approved by the Human Research Ethics Committee (University of the Witwatersrand, Johannesburg), Institutional Review Board of the Harvard T.H. Chan School of Public Health, The University of North Carolina at Chapel Hill Office of Human Research Ethics Biomedical Institutional Review Board, Johns Hopkins Research Project College of Medicine Research Ethics Committee, Joint Research Ethics Committee for the University of Zimbabwe, and Research Council of Zimbabwe. The focus group participants provided their written informed consent to participate in this study.

## Author Contributions

GD, DD, and SK conceptualized and designed the study. JL and NM conceptualized and established the processes, procedures, and tools to operationalize the adaptation steps of the ADAPT-ITT framework. JL, NM, GD, DD, and SK serve as members of the Adaptation Team who trained site personnel on how to operationalize the adaptation steps, oversaw completion of the steps, and reviewed adaptation feedback forms. JL and NM led the codification and categorization of adaptation feedback to inform integration of modifications into manuals and training agenda. JB, TK, PK, LM, TV, TC, and TN are site investigators and coordinators for the study who oversaw and implemented the adaptation steps on-site. GD, DD, SK, and NM participated in the first training on the intervention. JL, NM, GD, and DD prepared the first draft of the manuscript. All co-authors were involved in the manuscript review.

## Conflict of Interest

The authors declare that the research was conducted in the absence of any commercial or financial relationships that could be construed as a potential conflict of interest.

## References

[1] UNICEF. Children and AIDS. Statistical Update (2015). Available online at: https://data.unicef.org/resources/children-aids-2015-statistical-update-2/.2015

[2] Kharsany AB Karim QA. HIV infection and AIDS in Sub-Saharan Africa: current status, challenges and opportunities. Open AIDS J. (2016) 10:34–48. 10.2174/1874613601610010034 27347270 PMC4893541

[3] Francis SC Mthiyane TN Baisley K McHunu SL Ferguson JB Smit T . Prevalence of sexually transmitted infections among young people in South Africa: a nested survey in a health and demographic surveillance site. PLoS Med. (2018) 15:e1002512. 10.1371/journal.pmed.1002512 29485985 PMC5828358

[4] Fund UNP. The State of the World Population (2015).

[5] Melesse DY Mutua MK Choudhury A Wado YD Faye CM Neal S . Adolescent sexual and reproductive health in sub-Saharan Africa: who is left behind? BMJ Global Health. (2020) 5:e002231. 10.1136/bmjgh-2019-002231 32133182 PMC7042602

[6] Rani M Lule E. Exploring the socioeconomic dimension of adolescent reproductive health: a multicountry analysis. Int Fam Plan Perspect. (2004) 30:110–7. 10.1363/3011004 15381465

[7] Mandiwa C Namondwe B Makwinja A Zamawe C. Factors associated with contraceptive use among young women in Malawi: analysis of the 2015-16 Malawi demographic and health survey data. Contradept Reprod Med. (2018) 3:12. 10.1186/s40834-018-0065-x 30250748 PMC6146597

[8] Wado YD Bangha M Kabiru CW Feyissa GT. Nature of, and responses to key sexual and reproductive health challenges for adolescents in urban slums in sub-Saharan Africa: a scoping review. Reprod Health. 17:149. 10.1186/s12978-020-00998-5 32998741 PMC7526107

[9] Haas AD Technau C Pahad S Braithwaite K Madzivhandila M Sorour G . Mental health, substance use and viral suppression in adolescentsreceiving ART at a paediatric HIV clinic in South Africa. J Int AIDS Soc. (2020) 23:e25644. 10.1002/jia2.25644 33283916 PMC7720277

[10] Uthman OA Magidson JF Safren SA Nachega JB. Depression and adherence to antiretroviral therapy in low-, middle- and high-income countries: a systematic review and meta-analysis. Curr HIV/AIDS Rep. (2014) 11:291–307. 10.1007/s11904-014-0220-1 25038748 PMC4359613

[11] Wykowski J Kemp CG Velloza J Rao D Drain PK. Associations between anxiety and adherence to antiretroviral medications in low- and middle-income countries: a systematic review and meta-analysis. AIDS Behav. (2019) 23:2059–71. 10.1007/s10461-018-02390-8 30659424 PMC6639150

[12] Dow DE Turner EL Shayo AM Mmbaga B Cunningham CK O'Donnell K. Evaluating mental health difficulties and associated outcomes among HIV-positive adolescents in Tanzania. AIDS Care. (2016) 28:825–33. 10.1080/09540121.2016.1139043 26837437 PMC4905805

[13] Haas AD Ruffieux Y van den Heuvel LL Lund C Boulle A Euvrard J . Excess mortality associated with mental illness in people living with HIV in Cape Town, South Africa: a cohort study using linked electronic health records. Lancet Glob Health. (2020) 8:e1326–e34. 10.1016/S2214-109X(20)30279-5 32971055 PMC7582785

[14] Docrat S Besada D Cleary S Daviaud E Lund C. Mental health system costs, resources and constraints in South Africa: a national survey. Health Policy Plan. (2019) 34:706–19. 10.1093/heapol/czz085 31544948 PMC6880339

[15] Lund C Tomlinson M De Silva M Fekadu A Shidhaye R Jordans M . PRIME: a programme to reduce the treatment gap for mental disorders in five low- and middle-income countries. PLoS Med. (2012) 9:e1001359. 10.1371/journal.pmed.1001359 23300387 PMC3531506

[16] Chibanda D. The future of psychiatry in Africa-Thinking outside the box. Lancet Psychiatry. (2017) 4:741–2. 10.1016/S2215-0366(17)30368-1 28946947

[17] Demyttenaere K Bruffaerts R Posada-Villa J Gasquet I Kovess V Lepine JP . Prevalence, severity, and unmet need for treatment of mental disorders in the World Health Organization World Mental Health Surveys. JAMA. (2004) 291:2581–90. 10.1001/jama.291.21.2581 15173149

[18] Dorsey S Briggs EC Woods BA. Cognitive-behavioral treatment for posttraumatic stress disorder in children and adolescents. Child Adolesc Psychiatr Clin N Am. (2011) 20:255–69. 10.1016/j.chc.2011.01.006 21440854 PMC3088728

[19] Centers for Disease Control and Prevention. HIV/AIDS Prevention Research Synthesis Project: Compendium of HIV Prevention Interventions with Evidence of Effectiveness. Atlanta (1999).

[20] Wingood GM DiClemente RJ. The ADAPT-ITT Model: a novel method of adapting evidence-based HIV interventions. J Acquir Immune Defic Syndr. (2008) 47:S40–S6. 10.1097/QAI.0b013e3181605df1 18301133

[21] Donenberg G Fitts J Cohen M Ingabire C Olivier A Fabri M . Outcomes of the Kigali Imbreheza Project (KIP): a trauma-informed cognitive behavioral program to improve medication adherence and mental health among adolescents living with HIV in Rawanda (in preparation) (2020).

[22] Donenberg GR Cohen MH Ingabire C Fabri M Emerson E Kendall AD . Applying the Exploration Preparation Implementation Sustainment (EPIS) Framework to the Kigali Imbereheza Project for Rwandan Adolescents Living With HIV. J Acquir Immune Defic Syndr. (2019) 82 (Suppl 3):S289–S98. 10.1097/QAI.0000000000002204 31764266 PMC6884080

[23] Fabri M Cohen M Donenberg G. Trauma-informed cognitive behavioral therapy (enhanced) for HIV+ Youth in Rawanda Training Manual for Youth Leaders. Rawanda: The Kigali Imberehaza Project (2014).

[24] Cavanaugh CE Campbell J Braxton N Harvey J Wingood G. Adapting an evidence-based HIV-prevention intervention for women in domestic violence shelters. Psychol Violence. (2016) 6:469–77. 10.1037/vio0000042 27398257 PMC4933957

[25] Davis T DiClemente RJ Prietula M. Using ADAPT-ITT to modify a telephone-based HIV prevention intervention for SMS delivery: formative study. JMIR Formative Res. (2020) 4:e22485. 10.2196/22485 32831178 PMC7576465

[26] Khumsaen N Stephenson R. Adaptation of the HIV/AIDS Self-Management Education Program for men who have sex with men in Thailand: an application of the ADAPT-ITT framework. AIDS Educ Prev. (2017) 29:401–17. 10.1521/aeap.2017.29.5.401 29068714 PMC6477018

