# Supplementary material for: IMPAACT 2016: Operationalizing HIV Intervention Adaptations to Inform the Science and Outcomes of Implementation
Source: Front Reprod Health. 2021 May 28;3:662912. doi: 10.3389/frph.2021.662912 (PMC9580741; doi:10.3389/frph.2021.662912)

**Supplementary Figure 1.** Community Stakeholder Engagement Adaptation Feedback Form: (A) cover page with instructions to document stakeholder demographics, summary of adaptation recommendations, and documentation of Adaptation Team’s review and approval of modifications; and collection pages 2-4 documenting stakeholder responses and adaptation recommendations to open-ended questions for (B) clarity, (C) feasibility and acceptability, (D) barriers to understanding the manuals, and (E) barriers to delivering the group sessions.

**(A)**


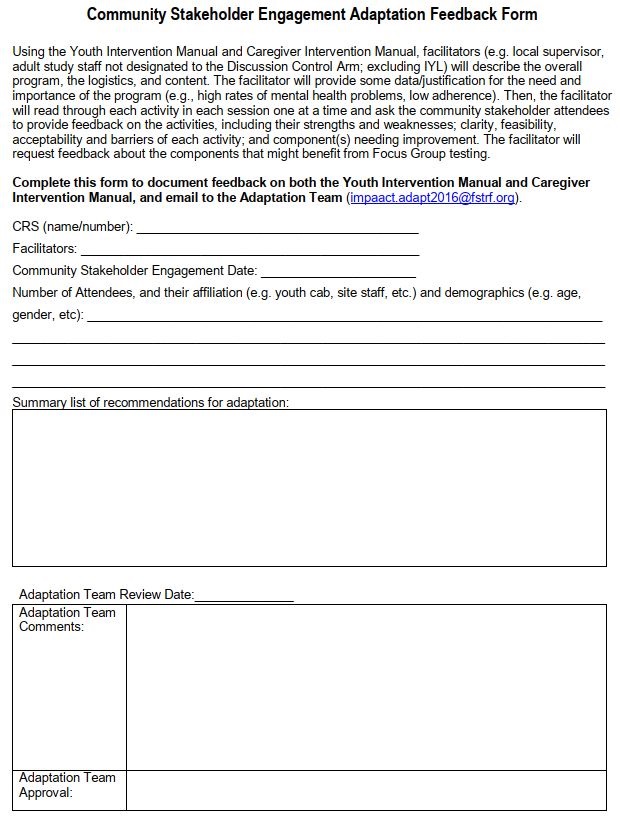


**(B)**


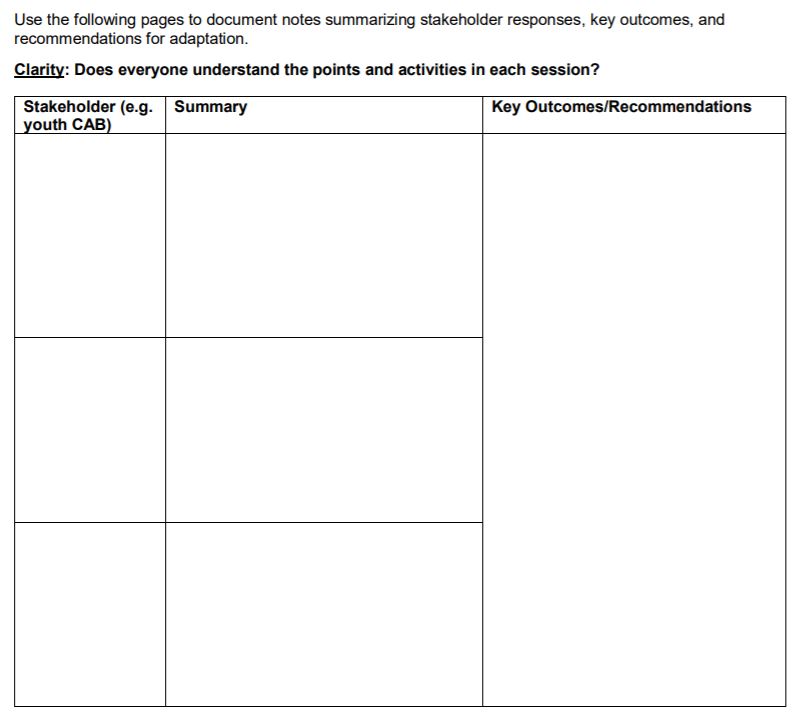


**(C)**


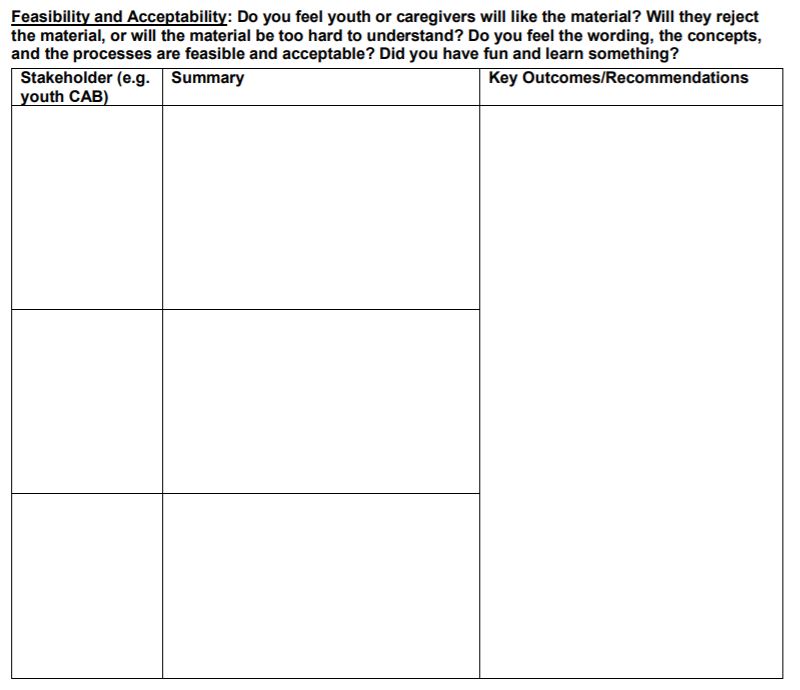


**(D)**


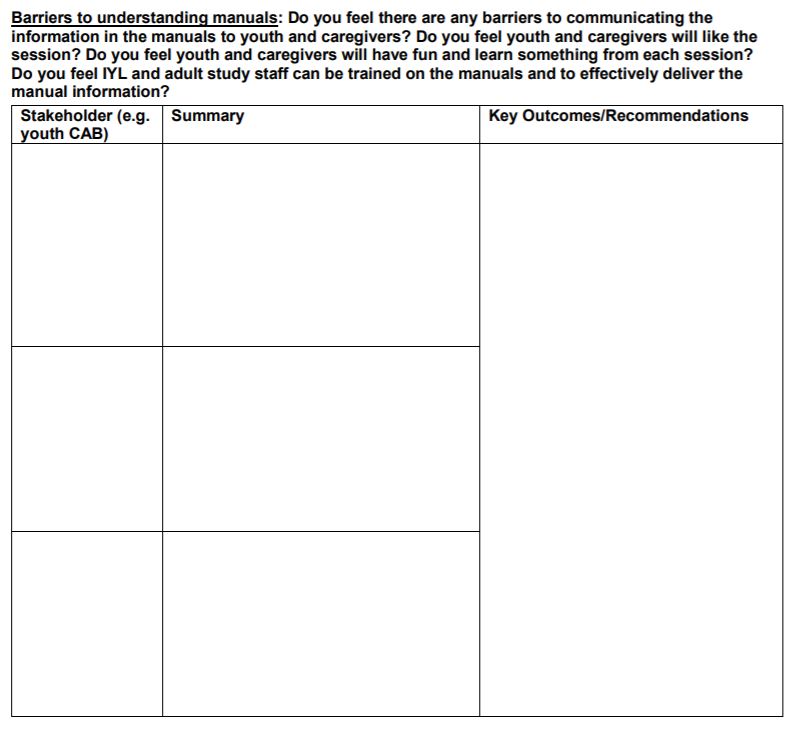


**(E)**


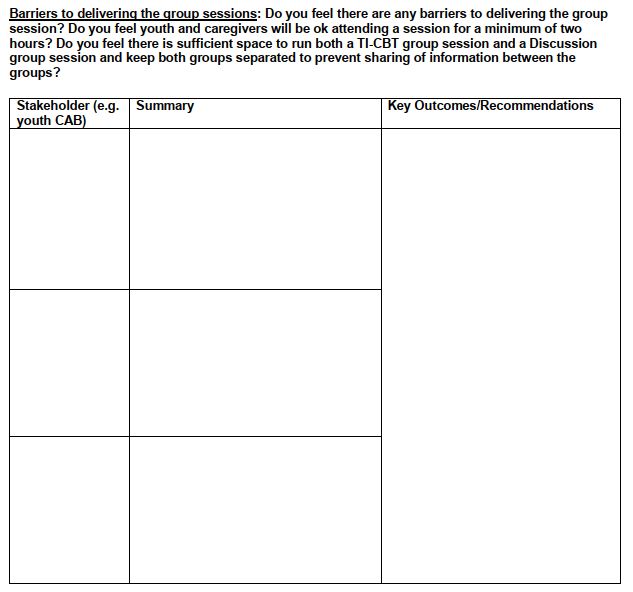


**Supplementary Figure 2.** Focus Group Adaptation Feedback Form: (A) cover page providing facilitators instructions and documenting stakeholder demographics, summary of adaptation recommendations, and documentation of Adaptation Team’s review and approval of modifications; and collection pages 2-3 documenting youth or caregiver responses and adaptation recommendations to open-ended questions for (B) acceptability and relevance and (C) other questions facilitators may have depending on the session delivered to participants.

**(A)**


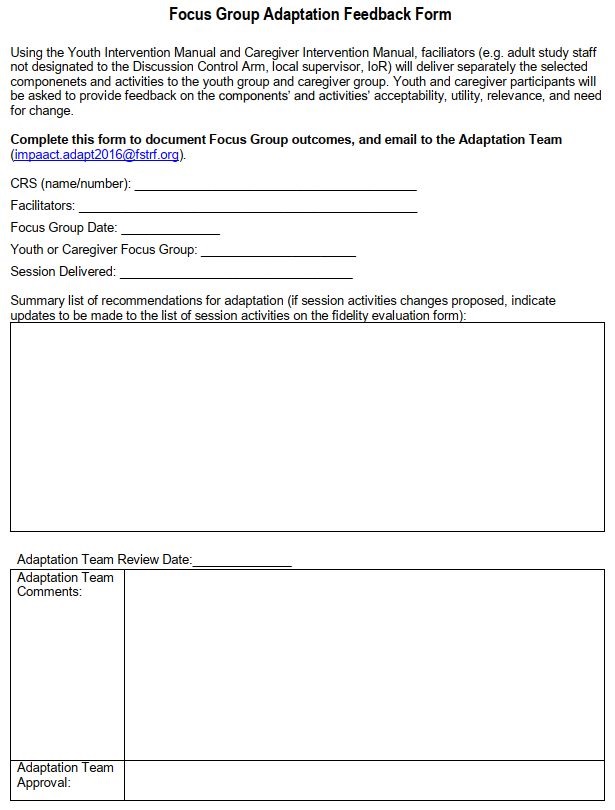


**(B)**


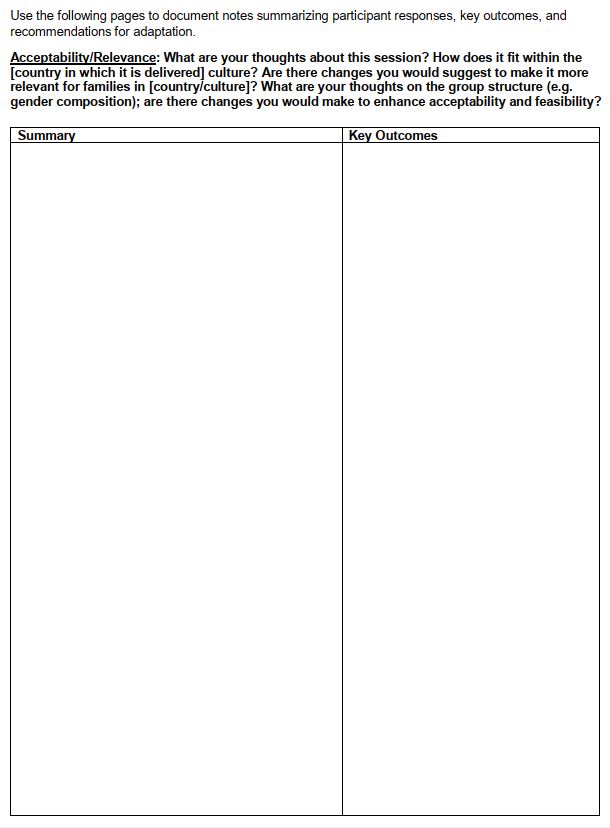


**(C)**


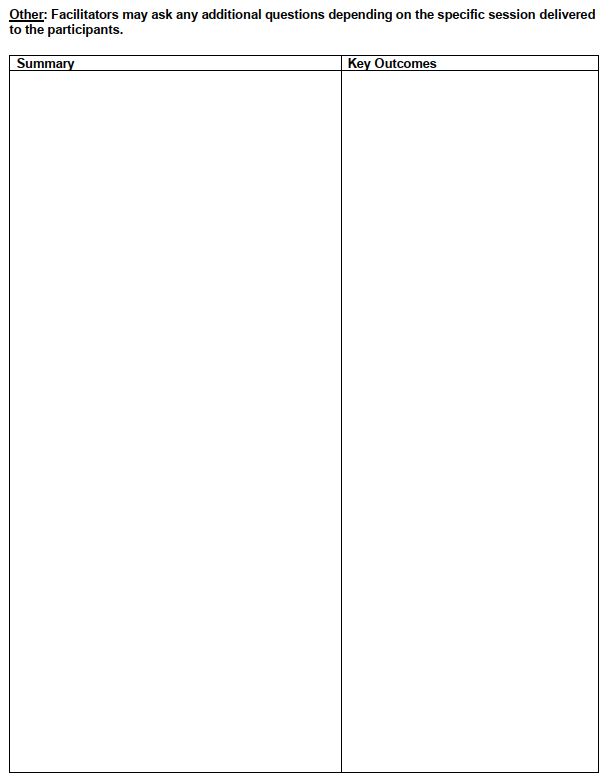

Supplement: Supplementary file 1 [file Data_Sheet_1.docx]
